# *Lactococcus lactis* Expressing Type I Interferon From Atlantic Salmon Enhances the Innate Antiviral Immune Response *In Vivo* and *In Vitro*

**DOI:** 10.3389/fimmu.2021.696781

**Published:** 2021-08-12

**Authors:** Carlos Muñoz, Josue González-Lorca, Mick Parra, Sarita Soto, Natalia Valdes, Ana María Sandino, Rodrigo Vargas, Alex González, Mario Tello

**Affiliations:** ^1^Laboratorio de Metagenómica Bacteriana, Centro de Biotecnología Acuícola, Departamento de Biología, Facultad de Química y Biología, Universidad de Santiago de Chile, Santiago, Chile; ^2^Laboratorio de Virología, Centro de Biotecnología Acuícola, Departamento de Biología, Facultad de Química y Biología, Universidad de Santiago de Chile, Santiago, Chile; ^3^ActivaQ S.A., Santiago, Chile; ^4^Laboratorio de Microbiología Ambiental y Extremófilos, Departamento de Ciencias Biológicas y Biodiversidad, Universidad de los Lagos, Osorno, Chile; ^5^IctioBiotic SpA, Santiago, Chile

**Keywords:** interferon Ia, *Lactococcus lactis*, antiviral, IPNV, Atlantic salmon

## Abstract

In salmon farming, viruses are responsible for outbreaks that produce significant economic losses for which there is a lack of control tools other than vaccines. Type I interferon has been successfully used for treating some chronic viral infections in humans. However, its application in salmonids depends on the proper design of a vehicle that allows its massive administration, ideally orally. In mammals, administration of recombinant probiotics capable of expressing cytokines has shown local and systemic therapeutic effects. In this work, we evaluate the use of *Lactococcus lactis* as a type I Interferon expression system in Atlantic salmon, and we analyze its ability to stimulate the antiviral immune response against IPNV, *in vivo* and *in vitro*. The interferon expressed in *L. lactis*, even though it was located mainly in the bacterial cytoplasm, was functional, stimulating Mx and PKR expression in CHSE-214 cells, and reducing the IPNV viral load in SHK-1 cells. *In vivo*, the oral administration of this *L. lactis* producer of Interferon I increases Mx and PKR expression, mainly in the spleen, and to a lesser extent, in the head kidney. The oral administration of this strain also reduces the IPNV viral load in Atlantic salmon specimens challenged with this pathogen. Our results show that oral administration of *L. lactis* producing Interferon I induces systemic effects in Atlantic salmon, allowing to stimulate the antiviral immune response. This probiotic could have effects against a wide variety of viruses that infect Atlantic salmon and also be effective in other salmonids due to the high identity among their type I interferons.

## Introduction

In aquaculture, the implementation of immunotherapies based on interferon administration to combat viral infections has been poorly studied. Aquaculture is one of the fastest-growing industries in the world. According to FAO estimates, in 2050 this industry is expected to be the main source of protein for human consumption (1). The main cultivated species include shrimp, tilapia, carp, rainbow trout, and Atlantic salmon, which due to high-intensity cultivation conditions, experience health problems caused by bacterial and viral pathogens, which reduce their productivity (2, 3). Among the main viral pathogens, we can find White Spot Syndrome Virus (WSSV) in shrimp (4), Tilapia lake Virus (TiLV) in Tilapia (5), and Koi Herpesvirus in Carps (6). In salmonids, several viral pathogens have been identified. In Atlantic salmon, the main viral pathogens are IPNV (7) and ISAV (8), which have shown devastating effects on the Chilean salmon farming industry (9). In rainbow trout, Atlantic salmon, and other salmonids, several emerging viruses have been identified; namely, VHSV, IHNV, SAV, and PRV, which show a lower impact on production (2). The main strategy used to date by salmon farming to prevent viral infections has been the use of vaccines; however, it has not been shown that they confer the same protection as that observed in mammals, requiring successive re-stimulations (boosters) to achieve efficient protection (10, 11).

In Atlantic salmon and other teleosts, the main response of the innate immune system against viral infections consists in the expression and secretion of Type I interferons (12), whose expression is induced as a consequence of the recognition of viral RNA by the cytoplasmic receptors RIG-I and MDA5, or by the membrane-associated receptors TLR3, TLR22 and TLR7 (13, 14). The interaction of type I Interferon with its membrane receptors stimulates the induction of a set of genes called Interferon Stimulated Genes (ISG), among which Mx and PKR are well known effectors (15–17). Mx is a GTPase structurally related to dynamin, which forms oligomers around the nucleoprotein, preventing virus transcription and replication (18). PKR is a kinase that inactivates translation initiation factor 2 by phosphorylation, preventing translation (19). In Atlantic salmon, 6 groups of type I Interferon have been identified, named from a to f (omitting g, to avoid confusion with gamma interferon) (20). Some of the first type I interferons from Atlantic salmon to be characterized were SasaIFN- α1 and SasaIFN-α2, both belonging to the current group Ia (IFN-Ia) (21). *In vitro*, recombinant SasaIFN-α1 exhibits strong antiviral activity against IPNV (14, 21), but has no effect on ISAV infections (22, 23). *In vivo*, administering recombinant SasaIFN-α2 increases resistance to IHNV infections (24), while administering intramuscular plasmids expressing IFN-Ia locally stimulates the expression of antiviral genes (25), and enhances the protective effect of DNA vaccines that express the HE proteins of ISAV (26). On the other hand, administering plasmids that allow the expression of IFN-Ic in fish confers protection against ISAV (25) and SAV (27). This background shows that the use of interferon I could be an effective and broad-spectrum tool to prevent mortalities caused by viral infections. However, its implementation involves developing an efficient administration system that avoids the need to inject each fish.

In mammals, lactic acid bacteria have been used successfully to synthesis and release immunoactive molecules such as interferons, antigens, or peptide hormones (28). The oral administration of these improved probiotics produces systemic effects, conferring protection against virus (29), bacterial pathogens (30, 31), and parasites (32). In salmonids, oral administration of *Lactobacillus casei* species expressing IPNV epitopes has shown to confer protection against the virus (33–37), while *Lactococcus lactis* strains have been used to orally immunize against Hirame novirhabdovirus in flounder (38), Carp spring viremia in common carps (39), the protozoa *Ichthyophthirius multifiliis* in Goldfish (40), and against the viral hemorrhagic septicemia in rainbow trout (41). Oral administration of recombinant *Lactobacillus casei* expressing epitopes of *Aeromona veronii* confers protection against this pathogen in *Cyprinus carpio* (42, 43), and *Carassius carassius* (44), while *Lactobacillus plantarum* expressing G protein of spring viremia of carp virus (SVCV) (45) and the ORF81 protein of koi herpesvirus (KHV) has shown confers protection against both viruses with high titers of IgM after its oral administration to *Cyprinus carpio* (46). Altogether, these results have shown that lactic acid bacteria are an efficient vehicle for the release of immunostimulant peptides in fish.

In this work, we explore the use of *L. lactis* to produce IFN-Ia from Atlantic salmon. Recombinant interferon was shown to have *in vitro* activity on CHSE-214 and SHK-1 cell cultures, increasing the expression of Mx and PKR, and reducing the production of viral particles in IPNV infection assays. *In vivo*, the administration of this probiotic was able to stimulate the expression of Mx and PKR in the spleen and head kidney, in addition to reducing the viral load.

## Materials and Methods

### Design and Synthesis of the P1-Usp45-IFNIa Module

To generate a module that allows the expression and export of Interferon I in *Lactococcus lactis*, a cDNA encoding for the IFN-Ia from *Salmo salar* (NM_001123710.1) between amino acid 24 and 175 was designed *in silico*, by *in frame* insertion of the sequence that encodes the peptide *Lactococcus lactis* Usp45 signal (GenBank: M35374), and incorporating the sequence of the constitutive promoter P1 from *Lactococcus lactis* (CP004884.1 1775802-1775887). The module was designed to be flanked by the NcoI and XbaI sites. The synthesis, codogenic optimization and cloning of this module (P1-Usp45-IFNIa) in the vector pUC57 was carried out by the company Genscript (http://www.genscript.com/).

By electroporation, plasmid pUC57/P1-Usp45-IFNIa was transformed into *E. coli* MC1061. Plasmid DNA was prepared from the transformants (FavorPrep ™ Plasmid DNA Extraction Mini Kit) and ~3 μg were digested sequentially, first with the XbaI enzyme (10 U), and then with the NcoI enzyme (10 U) in a final volume of 20 µl at 37°C for 1 hour. The released fragment was separated by agarose gel electrophoresis (Agarose 1%, TAE buffer), purified (FavorPrep ™ GEL/PCR Purification Kit) and used for its cloning in the vector pNZ8149 (Mobitec GmbH).

### Cloning of P1-Usp45-IFNIa in pNZ8149

Plasmid pNZ8149 was prepared in a similar manner to the P1-Usp45-IFNIa segment, ~ 3 µg of pNZ8149 DNA was treated first with XbaI (10 U), and then with NcoI (10 U) for 1 hour. The linearized plasmid was separated by agarose gel electrophoresis and purified using the FavorPrep ™ Plasmid DNA Extraction Mini Kit.

Purified P1-Usp45-IFNIa module and plasmid pNZ8149 from digestion with XbaI and NcoI were ligated with 400 U of T4 ligase (New England Biolabs) overnight at 4°C. The ligation was performed in an insert: vector ratio of 3: 1. Subsequently, the ligation was dialyzed against distilled H_2_O using a 0.02 µm dialysis membrane (Millipore), and electrotransformed into *L. lactis* NZ3900. The bacteria were subsequently plated in Elliker medium, supplemented with 0.5% lactose and cultured for 48 hours at 30°C. In this medium, positive transformants forms yellow colonies, since pNZ8149 contains as selectable marker the *lacF* gene which allows to *L. Lactis* NZ3900 grows using lactose as sole carbon source. The yellow colonies were separated, seeded on new Elliker plates, and analyzed by colony PCR, using primers pNICEF and pNICER (**Table 1**) to detect the presence of the insert. The PCR positive clones were again separated on Elliker plates, and the identity of the insert was corroborated by sequencing, after isolation of plasmid DNA from the recombinant *L. lactis* clones. This construction was named pNZ8149/P1-Usp45-IFNIa.

**Table 1:** Primers used for PCR and RT-qPCR.

Primer	Sequence
Mx-Fw	5’ TGT AAC ACG ATG CCC TCT CG 3’
Mx-Rv	5’ GAC GTC AGG GGA GCC AAT C 3’
PKR-Fw	5’ CAA TGA CCG ATT CCA GCT CC 3’
PKR-Rv	5’ CCC TTA TTT ATG CTAA TCC AG 3’
18S-Fw	5’ CCT TAG ATG TCC GGG GCT 3’
18S-Rv	5’ CTC GGC GAA GGG TAG ACA 3’
VP2F	5’ GAA GTC TTT CTG AGG TGG AGA G 3’
VP2R	5’ ATT CCT TTG GTC ACT AGT TGG T 3’
pNICEF	5´ TTA GAT ACA ATG ATT TCG TTC GAA GG 3´
pNICER	5´ CAA GCC TTG GTT TTC TAA TTT TGG 3´
pNZIFN-HisF	5´ TCT TAA TAA AGA ATT CAT AGT CTA GAG AGC TCA AGC 3´
pNZIFN-HisR	5´ TTA TTA AGA CGA ATT CTT AAT GAT GAT GAT GAT GAT GTC CAC CTC CAT ACA TTT GTG CAG CAA GAA T 3

### Insertion of the Spacer and Histidine Tail at the COOH end of IFN-Ia

To detect the recombinant expression of IFN-Ia (rIFN-Ia), the plasmid pNZ8149/P1-Usp45-IFNIa was modified by adding at the 3’ end of the IFN-Ia gene a sequence that codes for the GGGHHHHHH peptide. This sequence was incorporated by reverse PCR using the primers pNZIFN-HisF and pNZIFN-HisR (**Table 1**). The primer pNZINF-HisR hybridizes to the 3’ end of the interferon I gene (codogenically optimized), and replaces the stop codon with a sequence encoding the GGGHHHHHH peptide. After this sequence, the primer incorporates a stop codon and a site for the restriction enzyme EcoRI. On the other hand, the primer pNZINF-HisF hybridizes in the plasmid pNZ8149 in the region corresponding to the XbaI site and incorporates an EcoRI site at its 5’ end. After amplification, the PCR product was digested with the EcoRI enzyme, purified (FavorPrep ™ Plasmid DNA Extraction Mini Kit), and ligated with the T4-ligase enzyme. Subsequently, the ligation product was electroporated in *Lactococcus lactis* NZ3900. The identity of the new construct, prIFN-Ia, was corroborated by sequencing. The *Lactococcus lactis* strain NZ3900 containing the plasmid prIFN-Ia was named MT006.

### Preparation of *Lactococcus lactis* Electrocompetent Cells

The electrocompetent *Lactococcus lactis* NZ3900 cells was prepared based on the protocol suggested by Mobitec GmbH (47). A colony of *Lactococcus lactis* NZ3900 was inoculated in 5 ml of SG-GM17 medium (M17 medium containing 0.5 M sucrose, 2.5% Glycine, and 0.5% glucose) and cultured overnight at 30°C without agitation. The culture was inoculated in 40 ml of SG-GM17 medium and grown for 16 hours at 30°C, without shaking. The next day, this culture was inoculated in 400 ml of SG-GM17 medium and grown to an OD_600_ between 0.2 and 0.3. Subsequently, the culture was centrifuged at 6,000 x g for 20 min at 4°C and the collected pellet was washed three times with a cold wash buffer (0.5 M sucrose, 10% glycerol, 4°C). In each step, the pellet was collected by centrifugation at 6,000 x g for 20 min at 4°C and was resuspended by vortex in the corresponding buffer. After the last wash, the pellet was resuspended in 3 ml of wash buffer, aliquoted into 200 µl fractions, and stored at -80°C.

### MT006 Culture Conditions for Hybridization and Biological Activity Assays

From an isolated colony of MT006 (*Lactococcus lactis* NZ3900 prIFN-Ia), a pre-inoculum was prepared in 0.5% M17-Lactose medium; after incubating overnight, the culture was used to inoculate (2%) 40 ml of M17-Lactose 0.5% medium. After reaching an optical density at 600 nm between 0.6 and 0.8, the culture was induced with nisin 10 ηg/ml for 2 h. Bacteria were separated from the supernatant by centrifugation at 6,000 x g for 20 min at 4°C. The pellet obtained was used in western blot and biological functionality tests, while the supernatant was used in dot blot assays. The MT006 cultures were carried out at 30°C without shaking.

### Preparation of Cytoplasmic Extracts of MT006

From the MT006 culture, the bacterial pellet was resuspended in 1 ml of 1X PBS supplemented with 1 mM protease inhibitor PMSF. For their rupture, the resuspended cells were kept on ice and sonicated (ultrasonic processor, Sonic Vibracell) for 2 minutes, divided into 8 pulses (130 watts, 20KHz, 100%, 2 mm Cv188 stem) of 15 seconds with intervals of 1 minute. After treatment, the cellular debris was separated by centrifugation (13,000 x g, for 10 min at 4°C). The supernatant containing the cytoplasmatic proteins was removed and stored at -20°C. The total protein concentration was determined by the Bradford method.

### Preparation of Extracellular Protein Extracts From MT006 Cultures

The MT006 culture supernatant was treated with TCA (10% final), incubated on ice for 30 min, and then the proteins were precipitated by centrifugation at 9,000 x g for 30 min at 4°C. The obtained pellet was washed twice with acetone and dried at room temperature for 30 min. The pellet was resuspended in 1 ml of 1X PBS, supplemented with 1 mM PMSF. The total protein concentration was determined by the Bradford method.

### rIFN-Ia Detection in Cytoplasmic and Extracellular Protein Extracts From MT006 Culture

To detect rIFN-Ia in cytoplasmic extracts, the proteins were separated by mass using SDS-PAGE (Gel concentrator: 8% Acrylamide/Bisacrylamide 29:1, pH 6.8; resolutive gel: 10% Acrylamide/Bisacrylamide 29: 1, pH 8.8). 10 µg of total protein extracts from MT006 and MT005 (*Lactococcus lactis* NZ3900 pNZ8149) (control) were loaded together with the BenchMark ™ His-tagged Protein Standard. The samples were subjected to electrophoresis for 90 min at 100 V. After electrophoresis, proteins were electrotransferred (300 mA for 2 h at 16°C) to a nitrocellulose membrane. Subsequently, the membrane was blocked for 1 hour with a 2% BSA solution and washed 3 times with 1X PBS-Tween 20 (0.5%). The membrane was incubated with Rabbit polyclonal anti-His antibody (Abcam) (dilution 1/5000) for 1 hour at 37°C and washed three times with 1X PBS-Tween 20 (0.5%). It was then incubated with the polyclonal anti-rabbit IgG antibody conjugated to HRP (1/5000 dilution) for 1 hour at 37°C, followed by 3 washes with 1X PBS-Tween 20 (0.5%). The membrane was then incubated with 10 ml chemiluminescent developer solution (Pierce ™ ECL Western Blotting Substrate) and exposed to photographic film.

A dot blot was performed to detect rIFN-Ia in the extracellular protein concentrate. The nitrocellulose membrane was loaded with 10 µl of extracts to complete 1 µg of total protein per sample. Once air-dried, the membrane was hybridized and developed following the same protocol as described for the western blot.

### Quantification of the Amount of rIFN-Ia Produced by MT006

rIFN-Ia in cytoplasmic extracts was quantified by means of an ELISA test using 1 μg of protein extract. The plates were activated overnight at 4°C using 1X PBS buffer in the presence of the cytoplasmic extract. The wells were blocked with 2% BSA in 1X PBS buffer for 1 hour at 25°C. The first antibody, rabbit polyclonal anti-His Tag antibody (AbCam) dilution 1/5,000 in 1X PBS Tween 20 0.5%, was incubated at 37°C for 1 hour. The second antibody, goat polyclonal Anti Rabbit IgG conjugated to HRP (dilution 1/5000 in 1X PBS Tween 20 0.5%), was incubated for 1 hour at 37°C. Between each step, the wells were washed 3 times with 200 µl 1X PBS-0.5% Tween 20. The presence of bound antibody was determined by development with 100 µl of commercial TMB one-solution developer solution (Promega). After 15 minutes of incubation at 37°C, the reaction was stopped by adding 100 µl of 1N HCl. The product obtained was quantified by absorbance measurement at 450 nm. To quantify the concentration of Interferon present in the cytoplasmic extract, the following formula was used: rIFN-Ia (nM) = (OD450-0.0685)/0.0035, where IFN-Ia is the Interferon concentration in nM, and OD450 is the absorbance at 450 nm of the ELISA assay for rIFN-Ia.

### *In Vitro* Evaluation of the Immunostimulatory Activity of Recombinant Interferon

The determination of the immunostimulatory activity of the recombinant interferon was evaluated by the ability to induce the expression of the Mx and PKR genes. Both genes are part of the antiviral system activated by Interferon I and act as markers for this response.

Cultures of the CHSE-214 salmon embryo cell line were grown to 80% confluence in MEM supplemented with Fetal Bovine serum 2%, before treating them for 24 hours with different doses of recombinant interferon (10, 100 and 500 ηg/ml) from cytoplasmic extracts of MT006. The concentration of total proteins in each dose was equal to the protein concentration in the condition with the highest amount of rIFN-Ia (500 ηg/ml). Adjustment was achieved adding extracts of total protein from the *Lactococcus lactis* strain containing an empty pNZ8149 plasmid (strain MT005). PBS and cytoplasmic extracts of strains MT004 (*L. lactis* NZ3900) and MT005 were used as negative controls. As a positive control, cells were transfected with poly I:C (1 µg/ml), using 3 µl of FuGENE transfection reagent (Promega) following the instructions established by the manufacturer. After stimulation, cells were harvested, then total RNA was extracted using the E.Z.N.A. total RNA kit (Omega biotek), and quantification of the expression of Mx and PKR was performed using RT-qPCR. Each condition was assessed in triplicated (three independent experiments).

### Quantification of Mx and PKR Expression in Cell Cultures

Changes in the expression of Mx and PKR were quantified and evaluated by RT-qPCR. Once the RNA was extracted, its integrity was evaluated using agarose gels (1%, TAE 1X) and its amount determined by absorbance at 260 nm, using the Tecan Infinite 200 PRO equipment or a Synergy ™ 2.0 multi-well reader (Biotek). For each extraction the RT reaction was performed using 1 µg of total RNA and 100 Units of M-MLV reverse transcriptase (Invitrogen, USA) and 4 pmol of oligo-dT 18 mer. The RT reaction was performed first at 25°C for 10 minutes, then at 42°C for 1 hour, and finally stopped by denaturation at 65°C for 10 minutes. The qPCR reaction was developed using the SYBR Fast Universal qPCR kit (Kapa Biosystem USA), in 20 μl, using 2 μl of the RT reaction. To detect and quantify Mx, the primers Mx-Fw and Mx-Rv were used. The thermal program used consisted of 40 cycles of 15 seconds at 95°C, 15 seconds at 60°C, and 30 seconds at 72°C. PKR expression was quantified using the PKR-Fw and PKR-Rv primers (**Table 1**). A program similar to the one used with Mx was used for PKR, varying the melting temperature to 54°C. The 18S ribosomal RNA (18S-Fw and 18S-Rv primers, **Table 1**) was used to normalize the expression. The amplification program that was used consisted of 40 cycles of 15 seconds at 95°C, 15 seconds at 58°C and 30 seconds at 72°C. The qPCR reactions were performed in duplicate on a Stratagene Mx3000P kit. Each qPCR was performed in duplicated (technical replicates). The expression of the genes was normalized with regard to the control condition and the expression of the 18S gene using the ΔΔCt method described by Pfaffl (48).

### Evaluation of the *In Vitro* Antiviral Activity of Recombinant Interferon

To determine the effect of the recombinant interferon produced by *Lactococcus lactis* on the viral load of IPNV, cultures of the SHK-1 cell line (Sigma-Aldrich) were grown up to 80% confluence in L15 medium supplemented with 4 mM glutamine and 5% fetal bovine serum. These cultures were incubated with *Lactococcus lactis* cytoplasmic extracts containing 10, 100 and 500 ηg/ml of rIFN for 24 h. The total protein concentration was also equal to the condition where there is a greater amount of rIFN-Ia using protein extract of the control strain MT005. Poly I:C 1 µg/ml (final concentration) was used as a positive control, which was transfected according to the protocol indicated above. At the end of the incubation time, the infection was carried out at an M.O.I of 0.1 plaque-forming units per cell (PFU/cell). After 1 h of adsorption, cells were washed with MEM medium and placed again in MEM medium supplemented with 2% fetal bovine serum. The culture supernatant was extracted at 0, 6, 24, 48, 72, 120, and 168 hours post-infection for quantification of the viral load. Each condition was assessed in triplicated. The virus used in the assay correspond to an isolated belonging to the serotype Sp-2, genogroup 5.

### Quantification of Viral Load in Culture Supernatants

The viral load present in the culture supernatants of each well was determined under the following methodology. Total RNA was extracted from the supernatants using the EZNA total RNA kit (Omega Biotek). The RT and qPCR reaction was performed using the SYBR Fast One-Step qRT-PCR system (Kapa Biosystems), using 1 μg of total RNA, and the primers VP2F and VP2R (**Table 1**). To express the results in viral gene copy number, a calibration curve was established, based on the VP2 gene cloned in a pGEM-T vector. A range between 1×10^2^ to 1×10^8^ copies of the construct was used for the calibration curve.

### Preparation of IPN Virus for Challenge Tests

Infectious pancreatic necrosis virus (IPNV Sp-2) was propagated in monolayers of CHSE-214 cells grown in MEM medium supplemented with 2% fetal bovine serum. The infection was carried out at an M.O.I of 0.1 plaque-forming units per cell (PFU/cell). After 1 h of adsorption, cells were washed with MEM medium and placed again in MEM medium supplemented with 2% fetal bovine serum. Subsequently, it was incubated again at 16°C until the presence of a visible cytopathic effect was observed, approximately 48 to 72 hours post-infection.

The viral titer present in the culture supernatant was determined by the lysis plate method. Starting with the supernatant from the infected wells, serial dilutions were prepared with MEM medium, starting at from 10^-1^ upto 10^-11^. Subsequently, the dilutions of the supernatants were used to infect CHSE-214 cells. After 1 h of adsorption, cells were washed twice with PBS and kept for 72 hours in a semi-solid medium containing supplemented MEM and 0.5% w/v of agarose with low melting point. To fix the cells, 1 mL of formamide 37% v/v was added and incubated for another 30 minutes. To reveal the presence of lysis plaques, the agarose was removed, and 1 mL of crystal violet (1% crystal violet, 20% ethanol) was added to each well. After 30 minutes of incubation, excess crystal violet was removed, and the lysis plaques were quantified.

### Mixing *L. lactis* Strains with Fish Feed

The *L. lactis* strains (MT006 or MT005) were administered to the fish together with feed. The bacterial pellet of the cultures was washed with 1 ml of M17 medium, collected by centrifugation at 6,000 x g for 10 minutes at 4°C and resuspended in M17 medium in a volume equivalent to 1/10 of the volume of the original culture. The bacterial suspension was mixed with edible oil in a 2: 1 ratio and emulsified using a vortex. The emulsion obtained was mixed with the food and homogenized by shaking it in a plastic container.

### Evaluation of Immunostimulatory Activity *In Vivo*

The immunostimulatory activity *in vivo* was evaluated by determining the effect of the administration of the interferon-producing bacteria on the induction of the genes that express Mx and PKR in the main immunological organs of salmonids: spleen and head kidney. In the experiment, three groups of 15 fish (*Salmo salar* specimens of approximately 10 g each) were fed at 1% for 5 days. The first group was fed with food supplemented with 10^7^ CFU/(fish x day) of MT006, the second, with 10^7^ CFU/(fish x day) of MT005, and the third group received unsupplemented food.

Five fish of each group were sacrificed on days 1, 3, and 10 post-treatment. The spleen and anterior kidney immunological organs were extracted, collected in cryogenic tubes, and stored in liquid nitrogen. Total RNA was extracted from each immune organ, using TRISURE (Bioline) or the E.Z.N.A Total RNA kit (Omega Biotek) and the total RNA of each extraction was subsequently quantified by absorbance at 260 nm. RNA integrity was evaluated by agarose gel electrophoresis.

The RT-qPCR reaction was developed using the same procedure as that used to evaluate Mx and PKR expression in cell culture. The expression of Mx and PKR was analyzed in each organ of each fish sampled (five per condition). Each qPCR was performed in duplicated (technical replicates). Gene expression was normalized to the control diet consisting in food supplemented with MT005 and the expression of the 18S gene using the ΔΔCt method described by Pfaffl (48).

### *In Vivo* Effect of MT006 Administration on IPN Viral Load

Three groups of 10 fish (*Salmo salar*, 10* gr*) were used to evaluate the effect of oral administration of strain MT006 on the *in vivo* viral load of IPNV. One group was fed for 5 days at 1% of their weight, with food supplemented with 10^7^ CFU/(fish x day) of MT006. The other two groups functioned as controls and were fed at 1% with food supplemented with 10^7^ CFU of MT005 (group 2) and with food without supplementation (group 3). Once the treatment was finished, on day 6 the fish were infected intraperitoneally with 10^8^ PFU of IPN virus. Afterwards, the fish were sacrificed at 6, 25, and 60 days post-infection, and their immune organs, spleen, and head kidney were used to extract total RNA and perform RT-qPCR to quantify IPNV. Total RNA was extracted using TRISURE (Bioline) or the E.Z.N.A Total RNA kit (Omega Biotek). The integrity of the RNA was evaluated by agarose gels and its concentration was determined by absorbance at 260 nm. The RT-qPCR reaction was performed using 1 μg of total RNA and the SensiMix SYBR Hi-ROX One-Step kit (Bioline), under the same protocol described for the determination of viral load in cell culture.

### Fish Maintenance and Euthanize Protocols

The fish were acclimated for one week before treatment at 12°C in freshwater aquariums with a biomass not higher than 14 g/L, with continuous aeration, and fed with commercial pellets (EWOS MICRO™ 2 mm) at 1% of body weight. Water was maintained with a pH between 6.6 and 7, the salinity was adjusted to 6 PSU with NaCl to prevent fungal infection, and total ammonia was maintained in a range below 0.02 mg/L. Seventy percent of the water in all the aquariums was changed every day after feeding. Water parameters were monitored daily prior to and after changing the water. Feeding, changing the water, and measuring water parameters were all performed manually. The *L. lactis* strains (MT006 or MT005) were administered to the fish together with food.

To avoid unnecessary suffering of fish during the challenge and sampling, fish were anesthetized with benzocaine 40 mg/L for no longer than 2 min prior to the intraperitoneal injection, while sampled fish were euthanized with an overdose of benzocaine 40 mg/L, exposing fish during 5 to 10 minutes. Finally, fish were maintained in accordance with the ethical standards of the Institutional Ethics Committee of the Universidad de Santiago de Chile (approved in internal report n°350) and the relevant legislation in force.

### Phylogenetic Reconstruction and Bioinformatic Analysis

Evolutionary relationships between interferons encoded in the *Salmo salar* genome and that reported for SasaIFN-α1 was inferred using the Neighbor-Joining method (49) and the bootstrap test (1000 replicates) (50). The evolutionary distances were computed using the Poisson correction method (51) and are in the units of the number of amino acid substitutions per site. The rate variation among sites was modeled with a gamma distribution (shape parameter = 1). The ambiguous positions were removed for each sequence pair (pairwise deletion option). Evolutionary analyses were conducted in MEGA X (52). The amino acid sequences used are in the **Supplementary Table 1**.

The orthologous and paralogous sequences of Interferon Ia present in *Salmo salar* and other commercial salmonids species were identified using BLASTp against the genome of *Salmo trutta, Salvelinus alpinus, Oncorhynchus nekra, Oncorhynchus mykiss, Oncorhynchus kisutch*, and *Oncorhynchus keta.* As external groups we search also against the genome of *Esox lucius* and *Anguilla anguilla.* The minimum coverage cut-off was a 70%.

## Results

### Identification of Interferon Ia Sequence With Antiviral Activity Against IPNV

**Figure 1:**
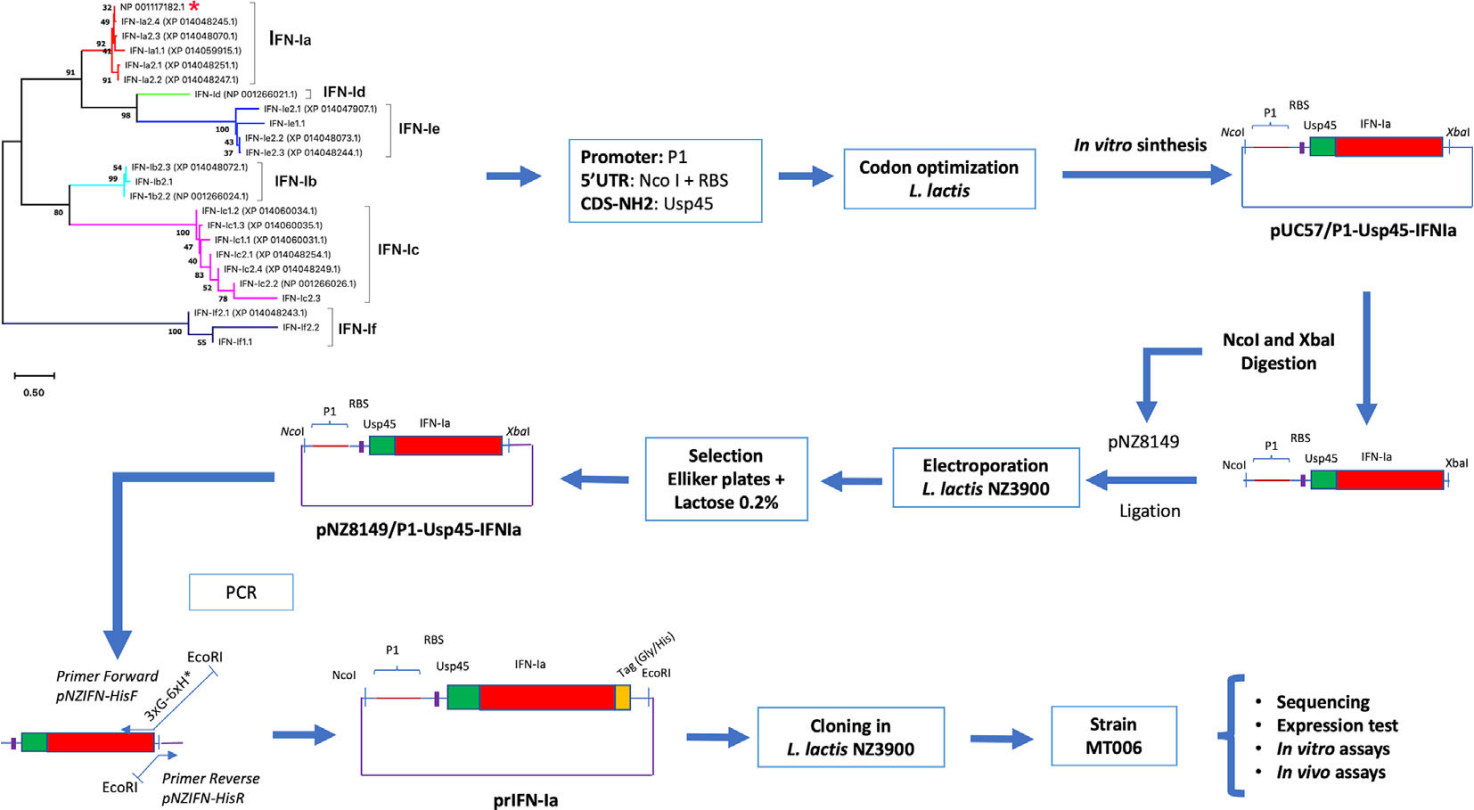
Experimental design to evaluate the use of *L. lactis* as an oral administration system in Atlantic salmon. The figure shows the experimental scheme to evaluate *L. lactis* as an Interferon oral administration system. The chosen Interferon Ia (red asterisk) was modified by incorporating a promoter (P1), the signal peptide of the Usp45 protein. The sequence was codogenically optimized and cloned into pUC57 (pUC57/P1-Usp45-IFNIa). This segment was cloned in pNZ8149, electroporated in *L. lactis* NZ3900 and selected on Elliker medium supplemented with 0.2% lactose. A tag (Gly/His) was added to the terminal COOH end of Interferon Ia by reverse PCR. The plasmid generated prIFN-Ia was cloned in *L. lactis* NZ3900 giving rise to the strain MT006. The recombinant constructs were sequenced and the functionality of rIFN-Ia was analyzed *in vitro* and *in vivo*.

The strategy outlined to evaluate the use of *L. lactis* as a release vehicle consisted of synthesizing *in vitro* a DNA segment that allows the constitutive expression of a gene that in its coding region allows in *L. lactis* the synthesis of recombinant interferon. On its amino-terminal end, this gene has the signal peptide of the protein Usp45, in its central region it encodes for interferon I from Atlantic salmon, and at its carboxyl end it encodes a tag for histidine that can be used for the detection of recombinant interferon (**Figure 1**). Once this synthetic DNA segment was obtained, it was cloned into the plasmid pNZ8149 and the production of the protein was evaluated *in vitro.* The antiviral activity *in vivo* and *in vitro* would be tested using IPNV for infection or challenge tests. As the Interferon I system of Atlantic salmon is constituted by six groups of genes, which show different degrees of antiviral activity, interest is focused on identifying a group with antiviral activity both *in vitro* and *in vivo*, which show functionality in its recombinant form produced in bacterial systems. Group Ia met these characteristics and, based on the work of Svingerud and collaborators (14), the sequence coding for Interferon Ia (SasaIFN-α1, NM_001123710.1, NP_001117182.1) was identified. Its classification as Interferon Ia was analyzed by phylogenetic reconstruction using interferon sequences from Atlantic salmon analyzed in the works by Liu and collaborators (20). The interferon encoded by NM_001123710.1 effectively clustered together with the IFN-Ia sequences encoded in the *Salmo salar* genome, indicating that the chosen sequence effectively encodes an IFN-Ia (**Figure 1**).

### Expression of Recombinant Interferon in *Lactococcus lactis* NZ3900

**Figure 2:**
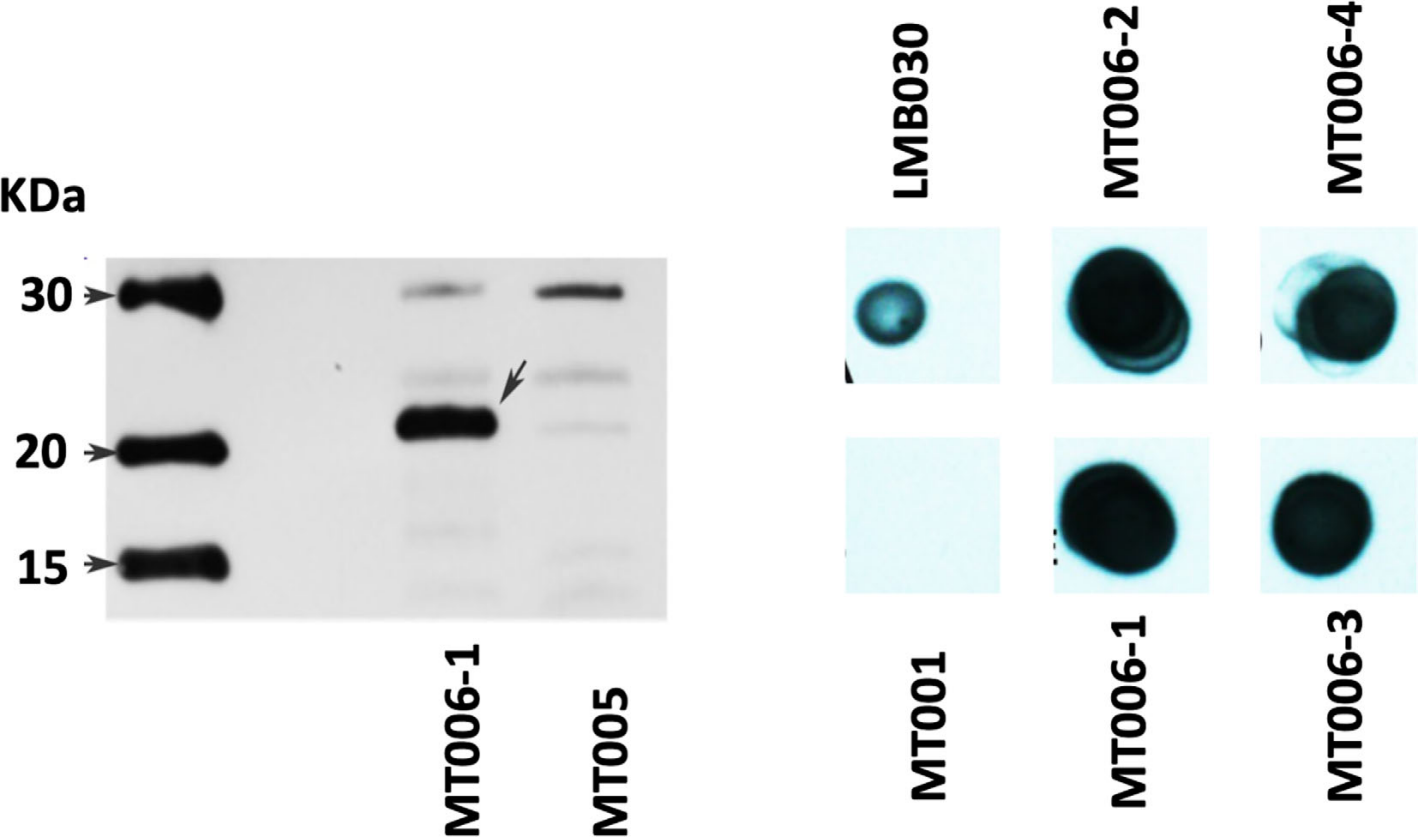
Expression of recombinant IFN-Ia in *L. lactis* NZ3900. On the left panel, the figure shows a western blot using cytoplasmic extracts of cultures of the strain MT006 (*L. lactis* NZ3900 + prIFNIa), and MT005 (*L. lactis* NZ3900 + pNZ8149). The arrow points to the recombinant interferon Ia (rIFN-Ia) in lane of MT006. The right panel shows a dot blot of protein extracts of the supernatants of the used cultures obtained by precipitation with TCA and levosucranase in the dot loaded with the extracts obtained from strain LMB030.

To determine if the designed interferon Ia gene would allow the expression of rIFN-Ia in *L. lactis*, the gene was cloned in the vector pNZ8149 and transformed into *L. lactis* NZ3900. The chosen clones were sequenced to corroborate the identity of the gene, and then their expression was evaluated by western blot from cytoplasmic extracts and the culture supernatant. The cytoplasmic extract of the MT006 culture induced with nisin showed a band of approximately 22 KDa, close to the 21.9 KDa theoretically estimated for its cytoplasmic form. This band is not present in cytoplasmic extracts of the MT005 strain, which corresponds to the wild type strain transformed with plasmid pNZ8149. When the western blot was repeated using protein extracts from the MT006 culture supernatant, a signal of the expected size was not observed (data not shown), which may be due to low concentration of the extracellular interferon. Proteins of the supernatant were then precipitated with TCA and a dot blot was performed loading approximately 1 µg of total protein. Four different clones were tested. The results show a positive signal in all four MT006 clones. No signal was observed in the culture supernatant of *L. lactis* strain NZ3900 containing plasmid pNZ8149 (MT005). As in the western blot, protein extracts of the LMB030 strain were used as a positive control, which expresses a 40 KDa protein that presents a histidine tag (**Figure 2**).

### Antiviral Activity of Cytoplasmic Extracts From *L. lactis* Expressing rIFN-Ia in Cell Cultures

**Figure 3:**
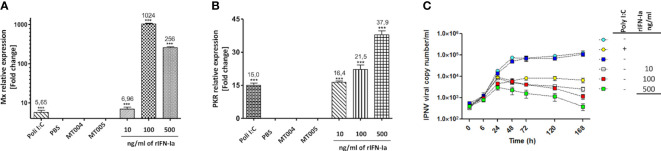
Antiviral effect of cytoplasmic extracts containing rIFN-Ia. The figure shows the effect of 10, 100 and 500 ng/ml of rIFN-Ia on the gene expression of Mx **(A)** and PKR **(B)** in cultures of CHSE-214. The CHSE-214 cultures were exposed to a constant concentration of total protein which was achieved by mixing the MT006 extracts with the MT005 extracts. As a control, the extract of the *L. lactis* NZ3900 (MT005) and *L. lactis* NZ3900 (MT004) strains were used. The expression of Mx and PKR was evaluated by RT-qPCR and expressed as normalized change times with regard to the expression of 18S rRNA and with respect to the cultures exposed only to MT005. The effect of rIFN-Ia on the viral replication kinetic **(C)** was evaluated by qPCR on the culture media of SHK-1 cultures exposed to 10 ηg/ml (open squares), 100 ηg/ml (red squares), and 500 ηg/ml (green squares) of rIFN-Ia, during the first 168 h post infection with IPNV. The SHK-1 cultures were exposed to a constant concentration of bacterial protein which was achieved by mixing the MT006 and MT005 extracts. As control SHK-1 cells were exposed to extract of MT005 (blue squares), transfected with Poly I:C (yellow circles), or remains without treatment (light blue circles). The existence of statistically significant differences was determined using a one-way ANOVA analysis (***p < 0.001).

To determine if the recombinant interferon produced by the MT006 strain shows bioactivity, CHSE-214 cell cultures were exposed to different amounts of rIFN-Ia (10, 100 and 500 ng/ml) for 24 hours. To keep constant the amount of total protein added to the CHSE-214 cell cultures in all conditions, the total amount of proteins was adjusted to the same concentration using cytoplasmic extracts of the MT005 control strain, which lacks the rIFN-Ia producing gene. The expression of Mx, and PKR was evaluated and also the effect of these extracts on the replication of IPNV in CHSE-214 cells. The results show that PKR expression increased in a dose-dependent manner (**Figure 3A**), while Mx reached its maximum induction (~1000 times) when cells were exposed to 100 ng/ml of extract from MT006 (**Figure 3B**). The induction of Mx and PKR at high concentrations of the MT006 extract exceeded the effect achieved by the Poly I:C transfection, indicating a specific effect achieved by the rIFN-Ia present in the MT006 extract.

Although several studies where the function of IFN-Ia from Atlantic salmon against IPNV has been tested in CHSE214 (14, 21), this cell line corresponds to an isolated from Chinook Salmon embryonic tissues. Thus, to properly test a more complex process such as resistance to IPNV infection in Atlantic salmon we change our cellular model to SHK-1 because is a cell line from Atlantic salmon. The SHK-1 was isolated from the head kidney of Atlantic salmon, and can be both infected and propagate IPNV Sp-2 (genogroup 5) (53, 54) in a mechanism dependent on macropinocytosis. However, to our knowledge, there is no direct evidence that Interferon Ia confers an antiviral state against IPNV infection, although several findings suggest it. SHK-1 increases the expression of Mx in response to Interferon Type I from rainbow trout (55), Poly I:C, and supernatant of Atlantic salmon macrophages stimulated with Poly I:C (56). In SHK-1, Poly I:C also induces the expression of Interferon Ia (57). To validate the use of SHK-1 to test the antiviral activity of extracts containing rIFN-Ia we first evaluated the effect of its transfection with Poly I:C on the expression of Mx, PKR, IFN-Ia, and the viral load of IPNV. Transfection with Poly I:C induced the expression of Mx, PKR and IFN-Ia between 10 to 30-fold in the first 24 hours and reduced the viral load two orders after 7 d post-infection (data not shown), indicating that the activation of the interferon type I pathway was effective to control the infection of IPNV in SHK-1. Based on these results we proceeded with the experiments.

When the effect on the replication of the IPN virus was analyzed in SHK-1 cells, it was observed that cells exposed to the extracts of MT006 present a lower viral load, reducing the number of copies of the VP2 gene by up to 2 orders of magnitude at the maximum amount of extracts of MT006. The exposure of SHK-1 cells to extracts of *L. lactis* strains that do not express rIFN-Ia had no effect on the number of copies of the VP2 gene, suggesting that the normal components of *L. lactis* do not induce an antiviral state. The effects achieved by the MT006 extracts on the viral load showed a dose-dependent behavior, reaching the maximum reduction at 500 ng/ml of rIFN-Ia. These antiviral effects were also greater than those observed when cells were transfected with poly I:C prior to infection with IPNV, suggesting that the rIFN-Ia present in the extract of MT006 has a highly specific antiviral effect on IPNV (**Figure 3C**).

### *In Vivo* Effect of the Oral Administration of MT006 on the Expression of Mx, PKR, and the Viral Load of IPNV

**Figure 4:**
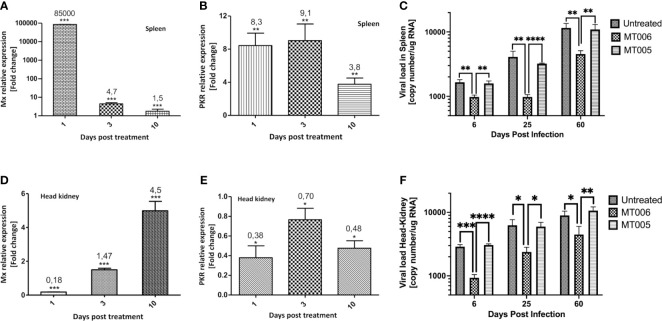
*In vivo* effect of the administration of MT006. The figure shows the effect of the administration of MT006 for 5 days [10^7^ CFU/(fish * day)] on the gene expression of Mx **(A, D)** and PKR **(B, E)** in spleen **(A, B)** and head kidney **(D, E)**. The effect on expression is shown as fold changes normalized to the expression of the gene encoding for 18S rRNA, and on expression in fish fed *L. lactis* NZ3900 containing plasmid pNZ8149 (MT005). The figure also shows the effect of MT006 administration on viral load in fish infected intraperitoneally with IPNV **(C, F)**. Viral load was normalized by the amount of RNA used in the RT-qPCR assay. The statistical analyzed was performed using a parametric t-test *p < 0.05, **p < 0.01, ***p < 0.001, ****p < 0.0001.

The results shown above indicate that extracts *L. lactis* expressing rIFN-Ia (strain MT006) stimulate the expression of antiviral genes (Mx and PKR) in CHSE-214 and reduce the viral load in the supernatant in SHK-1 cell cultures, suggesting that this strain could help control infections by IPNV or other viruses sensitive to IFN-Ia. To evaluate the functionality *in vivo*, the effect of the administration of MT006 for 5 days (10^7^ CFU/(fish×day)) on the expression of Mx and PKR, and on the viral load in fish challenged with IPNV, was evaluated on 1, 3 and 10 days after the end of the treatment (fed) with MT006. The results show that the effect of administering MT006 is mainly on the spleen, where Mx reaches a maximum induction of 85,000 times the first day after the end of treatment with MT006 (**Figure 4A**), while PKR reaches an increase of around 8 to 9 times on days 1 and 3 post-treatment (**Figure 4B**). In the case of the head kidney, an inverse behavior to that observed in the spleen was noted, where a reduction of the expression is observed in the head kidney on the days of maximum induction (**Figures 4D, E**). To our knowledge, this striking behavior has not been previously described in salmonids, however the stimulation of fish with Poly I:C and R848 induce the expression of IFN-Ia mainly in the spleen, suggesting that this cytokine is related with the immune function of this organ (14). These data were normalized with respect to the fish fed with MT005, analyzing only the changes in expression consequence of the rIFN-Ia expression. When we analyze if *L. lactis* perse could increase the expression of Mx and PKR in the spleen or kidney, we also observed a different pattern of stimulation in both organs. While in the spleen MT005 increased the expression of Mx (25-fold) and PKR (11-fold) only at 10 days post-treatment (**Supplementary Figures 1A, C**), a faster response was identified in the kidney that increased the expression of Mx (7.7 fold) and PKR (5 fold) only on the first day post feed with MT005 (**Supplementary Figures 1B, D**). Altogether these results showed that the spleen was the main target of the rIFN-Ia produced by MT006, showing a faster and strong response than the kidney. While the bacterial host (*L. lactis* NZ3900) induced an antiviral response weaker and later to that induced by MT006, targeting first to the kidney and later to the spleen.

When the effect of the administration of the probiotic expressing rIFN-Ia (MT006) was analyzed in challenge assays with IPNV, it was observed that fish treated with MT006 showed a lower viral load than fish treated with *L. lactis* NZ9000 or without treatment both in spleen as in head kidney (**Figures 4C, F**). This effect was observed in the 3 timepoints analyzed (6, 25 and 60 days post-infection), although the magnitude of this effect decreased towards the end of the experiment, suggesting a long-term effect in increasing the antiviral capacity induced by MT006. Mortality was not detected in the fish challenged with the pathogen, in agreement with the current presence of QTL IPNV-resistant fish in Chilean salmon farming centers that results in a high prevalence of the infection with a lower mortality.

### Potential Antiviral Effect of MT006 on Other Salmonids

**Figure 5:**
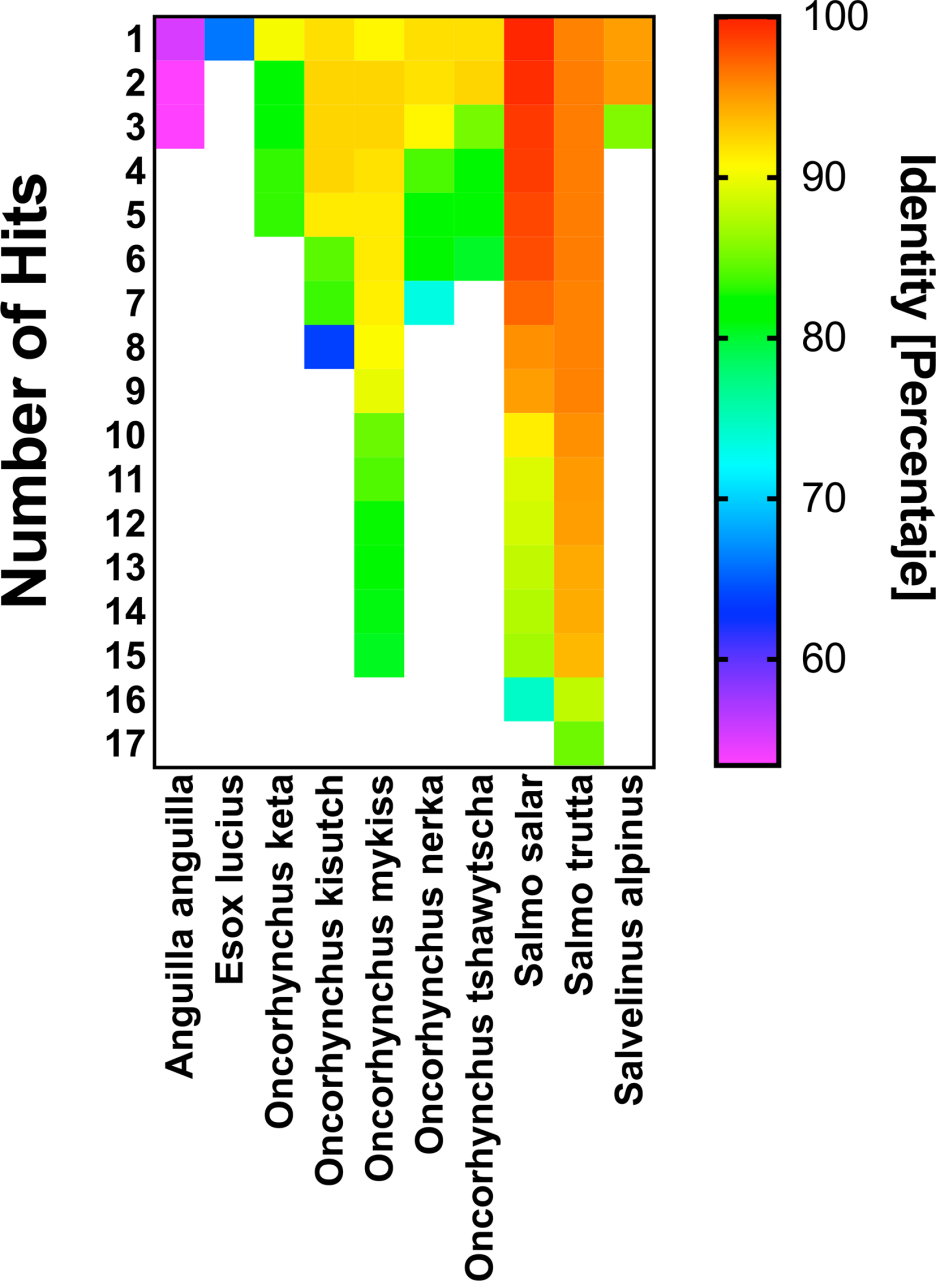
Comparison of rIFN-Ia with interferons present in other Salmonids. The figure shows the percentage of identity of rIFN-Ia with interferons present in the salmonid species *Salmo trutta*, *Salvelinus alpinus*, *Oncorhynchus nekra*, *Oncorhynchus mykiss*, *Oncorhynchus kisutch*, *Oncorhynchus keta*, and in other fish (Eagle eel and Esox lucius). The figure shows the number of hits identified in each species and the percentage of identity. Both data were obtained by BLASTP alignment against database nr. The identity percentage is shown in a color scale and was constructed with the Prism 9.0 program.

Our results show that the rIFN-Ia produced by MT006 has *in vitro* and *in vivo* effects on the antiviral activity of *Salmo salar*, where the administration of the strain MT006 suffices to confer an antiviral state that reduces the load of IPNV. The application of MT006 in other salmonids will partially depend on the degree to which rIFN-Ia from *Salmo salar* can stimulate IFN-I receptors. To evaluate the potential effect of MT006 on other salmonids, it the protein sequence of rIFN-Ia was compared with the sequence of interferons previously identified in other salmonids. We identified genes that encode for proteins with over 90% identity to rIFN-Ia in Salmo trutta, *Salvelinus alpinus*, *Oncorhynchus nekra*, *Oncorhynchus mykiss*, *Oncorhynchus kisutch*, and *Oncorhynchus keta*, with higher identity in Interferons I of *Salmo trutta* and *Salvelinus alpinus* (**Figure 5**). These results suggest that oral administration of MT006 to other salmonids could help reduce the impact of viral infections in these species.

## Discussion

The microorganisms that conform the microbiota play an important role in the adequate stimulation of the immune system (58). Although this interaction has mainly been characterized in mammals, the common characteristics shared by the immune system of mammals and teleost fish, in addition to the similarities in the complexity of the microbiota that is established in the intestines and mucosa in both groups, suggest that the mechanisms of communication are conserved (59). Experiments using zebrafish as models have established the role of the microbiota in the maturation of the immune system (60), protection against pathogens (61), nutrient uptake (62), behavior (63), and in the formation of bones (64). In salmonids, although progress has been made in characterizing the composition of the microbiota in various environments and how it is affected by the culture and feeding conditions, there are few studies that show a direct relationship with the immune system (65–69). Indirect evidence has been obtained regarding the effect of probiotics on the immune system of salmonids, where in *Salmo salar* and *Oncorhynchus mykiss*, have been shown to stimulate the innate immune response against bacterial and viral pathogens (70). The mechanisms that allow the interaction between the immune system and the microbiota have not been fully clarified. The evidence obtained so far indicates that its communication is mediated by the interaction of structural components of microorganisms with PRR or host antibodies, and by molecules secreted by microorganisms that are detected by the immune system, in a mechanism similar to that used by the endocrine system (58, 71). The presence of these molecules in the microbiota of fish and mammals, as well as their receptors in their respective hosts (59), suggest that the communication mechanism arose early in evolution and has been conserved and improved in the various species (72).

The present work exploits this communication capacity of the host with its commensal microorganisms using a probiotic bacterium *L. lactis* as a vehicle for the production and release of rIFN-Ia in *Salmo salar* to stimulate the antiviral response of the fish. Moreover, *L. lactis* have been identified as normal components of the microbiota of mammals and salmonids (73, 74). Similar strategies have been used in mammals, allowing to stimulate the anticancer response through the secretion of IL-17A (75), stimulating the adaptive immune response by secreting IL-12 (76), or reducing the intestinal inflammation by secreting IL-10 (77), or IL-35 (78). The expression of functional proteins from animals using bacterial systems lacking the post-translational modification machinery is not always feasible when these modifications are necessary for the proper folding of the protein or for recognition by its receptor. In the case of cytokines, some of them, such as type I and II interferons in humans, are glycosylated or have glycosylation motifs (uniprot.org). The functionality of human type I interferon expressed in *E. coli* (79) and *L. lactis* (80) suggests that glycosylations are not related to the interaction with its receptor. However, they participate by increasing the half-life of the protein, stabilizing the 3D structure, or protecting it from the action of proteases (81, 82). In the case of Interferon Ia from *Salmo salar*, its glycosylated isoforms have not been described despite the fact that it also presents glycosylation motifs in its primary sequence. As in the case of Interferon I of humans, the expression of recombinant interferon Ia from *Salmo salar* in its biologically active form has also been successfully achieved using *E. coli* as a recombinant protein expression system (83), supporting our observation that Interferon Ia from *Salmo salar* is also biologically active in its non-glycosylated form.

Our original design involves modifying the primary sequence of *Salmo salar* IFN-Ia by introducing the signal peptide of the USP45 protein. This peptide has been widely used in order for *L. lactis* to secrete proteins to the extracellular medium (84). However, the efficiency of this signal appears to depend on the recombinant protein. In the case of human interferons, the signal peptide of the USP45 protein produces an inefficient secretion, just as we observed in the case of salmon rIFN-Ia presented in this work. This efficiency has been improved by incorporating additional signals to the USP45 protein peptide (85). It remains to be determined whether these signals could also increase the export efficiency of rIFN-Ia. However, according to our results, this inefficient secretion is sufficient to produce biological effects *in vivo*. This is consistent with the low concentrations of IFN-Ia in serum (100-1000 pg/mL) that are observed *in vivo* in response to viral infections (86) or during the administration of interferon in hepatitis C treatments (87).

The mechanism by which the *in situ* release of these cytokines at the mucosal level produces local and systemic effects is not fully understood, but it is estimated that the release of these cytokines would stimulate the immune cells associated with the mucosa, and these, in turn, would amplify the effect when translocated into lymph nodes. An important difference in the organization of the immune system of fish and mammals is that the former have diffuse mucosal-associated immune tissue, without the presence of lymphoid nodes (88). Therefore, if the first suggested mechanism operates, it would imply that rIFN-Ia could stimulate the immune cells present in the intestinal mucosa or be phagocytosed by them, producing the migration of these immune cells to the spleen or kidney. There, the immune cells could stimulate other cells either by endogenous release of interferon or by the release of interferon produced by MT006, in a mechanism similar to that observed when dendritic cells interact with commensal bacteria in the mammalian intestine and transport them to the lymph nodes (89). This mechanism could explain the immunizing properties of orally administered recombinant Lactic Acid Bacteria that express epitopes from microbial pathogens of fish (33–44). The dosages used in these experiments are between 10^7^ to 2x10^8^ CFU per fish gram, around 30 to 600 times the used dosage of MT006. This high dosage allows reduce the spleen viral load of IPNV 32 times (37), far more than our results that show a reduction of 3 -4 times, in the first days after the challenge. It remains to determine whether longer treatment with MT006 has the potential to reduce the viral load to levels similar or lowers at the observed with the immunizing *Lactobacillus casei* expressing the epitopes of VP2 of IPNV (37). The characterization of the immune response at the gut level during and after the administration of MT006 should help to clarify if this proposed mechanism plays a role in the immune stimulation produced by the *Lactococcus lactis* strain expressing rIFN-Ia.

An alternative mechanism that could explain the systemic effects of orally administered probiotics could be the spread of these bacteria to different tissues. The translocation of bacteria from the gastrointestinal tract to other organs has been observed mainly under pathological conditions where inflammatory processes increase the permeability of the epithelial barrier. However, this has also been observed under physiological conditions (90). The translocation of *L. lactis* from the intestine to internal organs such as the mammary glands has been identified in pregnant female mice shortly before giving birth (91). Interestingly, IFN-I promotes the integrity of the gastrointestinal barrier mediated by a reduction in apoptosis of epithelial cells (92). Thus, if IFN-Ia has the same effect in fish, it is unlikely that rIFN-Ia secretion promotes the translocation of MT006. However, it cannot be discarded as a mechanism since there are several examples of symbiotic relationships between fish and bacteria based on the colonization of internal and external organs (93–96). On the other hand, the presence of bacteria in the liver and head kidney has also been detected in healthy wild fish (97), a situation that indicates that the relationship that fish have with their commensal bacteria is more complex than that observed in mammals.

The *in vivo* stimulation with MT006 also showed that the spleen and kidney have an opposite kinetics of Mx, being the spleen, the organ/tissue preferentially stimulated at a short time after treatment with MT006. To our knowledge, this behavior has not previously described, probably because most of the studies that analyze *in vivo* the effects of IFN-I have been conducted analyzing the expression in one organ at different times or comparing both organs at the same time. The preferential stimulation of the spleen could be explained if these organs express receptors specific for IFN-Ia which expression should be regulated at the translational level, since both organs show no differences in the transcription of IFN-Ia receptors (98). By other hand, the reason why the spleen is more sensitive to INF-Ia could be related to its function as a secondary immune organ related to the antibody production by B-cells, which is improved in Atlantic salmon by IFN-I (26). In relation to the opposite kinetics of Mx expression observed in the spleen and kidney, the reduction of Mx expression in the spleen cloud be the result of the lost MT006 in the intestine, while the increased expression of Mx in the kidney could be explained if the kidney responds to a secondary signal emitted by the spleen. Since interferon type I can stimulate its own expression, the rIFN-Ia could induce the expression of Interferon Ic or Ib which are able to produce systemic effects. It remains to determine whether Interferons type I shows a cross-stimulation pattern among them.

Our results showed that administration of MT006 before the infection with IPNV reduced the replication of the virus but was not able to help at resolves completely the infection, which continued achieving after 60 days a viral load like those observed on day 6. Continuous treatment with MT006 higher than five days could be useful to improve the capacity of fish to completely resolve the infections of IPNV, especially in fish with QTL resistant to the mortality but not to the infection, such as the fish cultured in Chilean salmon farming centers. Since IPNV has shown an immunosuppressive effect on fish (99), a reduction in viral load mediated by the oral administration of MT006 should help to improve the robustness of fish against co-infection with pathogens either bacterial or viral (100).

Interferon Ia has shown antiviral effects against IHNV (24), IPNV (83), SAV (101) and an adjuvant effect in vaccines against ISAV (26), a situation that allows us to suppose that the administration of MT006 could also have antiviral effects against SAV or act as adjuvant of ISAV vaccines in *Salmo salar* or other salmonids infected by these viruses, which possess a system of Interferons with a high percentage of identity (>90%) with the rIFN-Ia produced by MT006. For example, IPNV, and SAV can also infect *Salmo trutta*, and *Oncorhynchus mykiss* (7, 102–104); therefore, they could constitute species in which to evaluate the antiviral effect of the administration of MT006. This implies that MT006 could be used as a broad spectrum biotherapeutic agent in salmonid aquaculture, either to induce an antiviral state, or to enhance the effect of vaccines against various bacterial or viral pathogens that affect salmon farming.

## Conclusions

The results obtained in this work indicate that *L. lactis* is a suitable vehicle to produce Interferon Ia from *Salmo salar* in its biologically active form and that oral administration of this rIFN-Ia producing bacterium stimulates the systemic antiviral response in fish, enabling a reduction in the viral load in immune organs.

Our work supports the use of *L. lactis* as a vehicle to specifically stimulate the immune response in teleost fish through the production and/or secretion of immunostimulating peptides.

## Data Availability Statement

The raw data supporting the conclusions of this article will be made available by the authors, without undue reservation.

## Ethics Statement

Experiments with fish were designed and done in accordance with the ethical standards of the Institutional Ethics Committee of the Universidad de Santiago de Chile (approved in internal report n°350) and the relevant legislation in force.

## Author Contributions

Conceptualization, MT, CM and JG. Methodology, CM, MP, and JG. Investigation, CM, SS Resources, AS. Writing—original draft preparation, AG, R.V, NV. Funding acquisition, MT. All authors contributed to the article and approved the submitted version.

## Funding

This research was funded by Fundacion Innovación Agraria (FIA), grant number PYT 2012-0056 to MT. The funder was not involved in the study design, collection, analysis, and interpretation of data, the writing of this article or the decision to submit it for publication.

## Conflict of Interest

Authors MT and AS were employed by the company IctioBiotic SpA and ActivaQ S.A., respectively.

The remaining authors declare that the research was conducted in the absence of any commercial or financial relationships that could be construed as a potential conflict of interest.

## Publisher’s Note

All claims expressed in this article are solely those of the authors and do not necessarily represent those of their affiliated organizations, or those of the publisher, the editors and the reviewers. Any product that may be evaluated in this article, or claim that may be made by its manufacturer, is not guaranteed or endorsed by the publisher.
