# Supplementary material for: Lactococcus lactis Expressing Type I Interferon From Atlantic Salmon Enhances the Innate Antiviral Immune Response In Vivo and In Vitro
Source: Front Immunol. 2021 Aug 12;12:696781. doi: 10.3389/fimmu.2021.696781 (PMC8406758; doi:10.3389/fimmu.2021.696781)
Supplement: Supplementary file 1 [file DataSheet_1.docx]

Supplementary Material

## Supplementary Figures


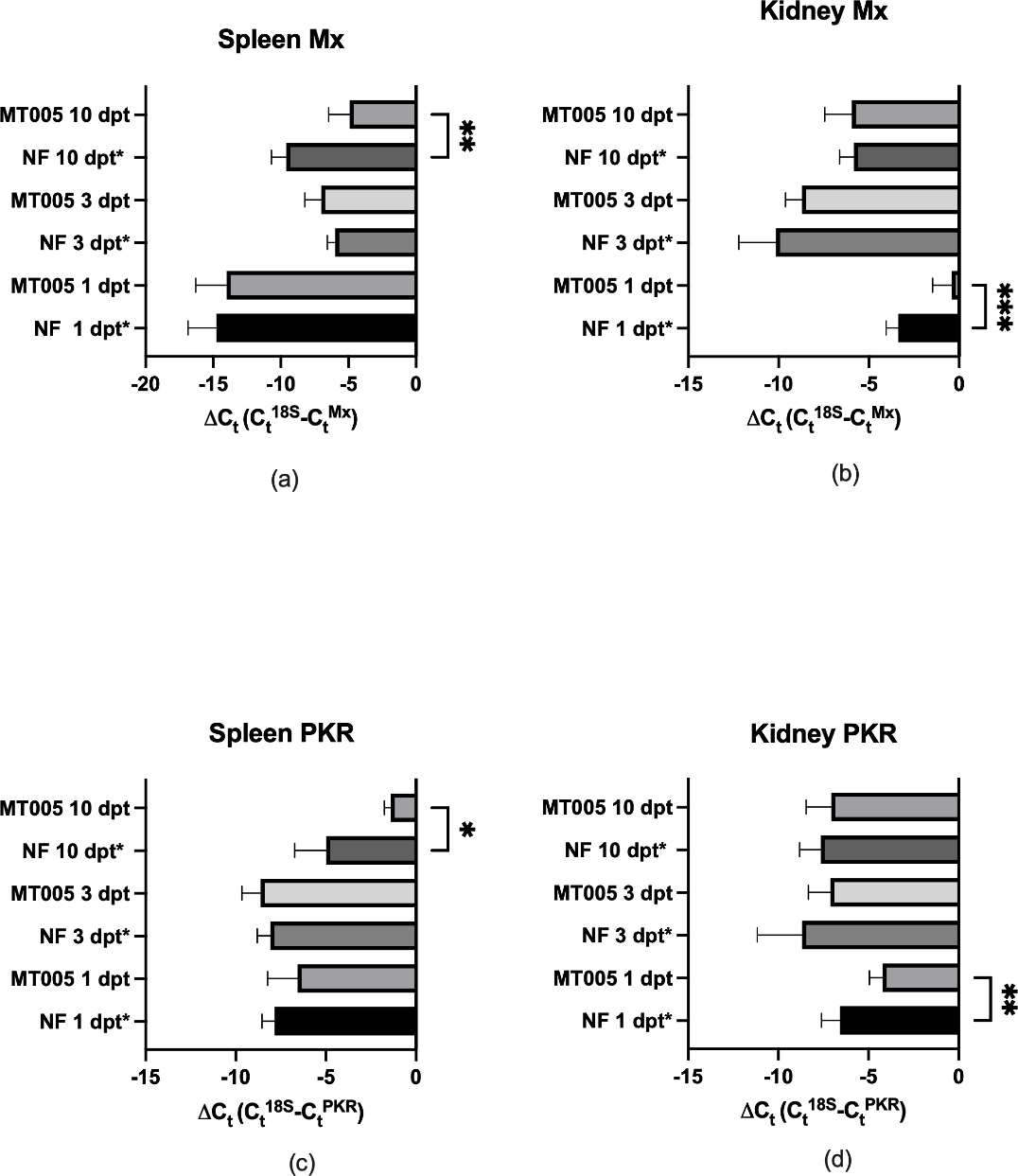


**Supplementary Figure 1**. The figure shows the difference between the Ct obtaining amplifying the 18S ribosomal RNA (C_t_^18S^) and the Ct obtained amplifying the Mx (C_t_^Mx^) and PKR (C_t_^PKR^) genes using total RNA extracted from the spleen or kidney of fish treated during five days with normal feed (NF), *L. lactis* pNZ8149 (MT005) collected at 1, 3 and 10 days post treatment (dpt) with MT005 or at the same time in the case of fish fed only with normal feed (dpt*). The significance was analysed using a t-test (* p< 0.05), ** p< 0.01, *** p< 0.001).

## Supplementary Tables

**Supplementary Table 1**

**Amino acid sequences of Interferons type I described in Atlantic Salmon**

| **Identifier** | **Amino acid sequence** |
| --- | --- |
| >IFNIa2.1 (XP_014048251.1) | MYTMQSWTCICLIICSMQSVCHCCDWIQHHYGHLSSEYLSLLDQMGGDITEQDAPVFFPTSLYRHIDDAGFEDQVRFRNETIYQITKLFDGNMKAVTWDKKKRDDFLNILERQFENLKSCVSPAKKPERRLKRYFKELNRKVLRKMNYSAQAWELIRKETRRHLQRLDIFKAKIH |
| >IFNIc2.2 (NP_001266026.1) | MALQTITWMSAFLCVAHVCSMPMPCQLQGQLVRITHNLLRDMGGNFPLECLQENVFVAFPATAFASSGAPQLGSSGAKAIYETLKNIDILFEADDLPTQWDQQKLKNFQNIVYRQIEESKCMMGSVDTSDYLIRTEGLNTYFGNIAAVLKEKNFSYCAWEVVRKELLYTLQFILEHNSDSLLWANRT |
| >IFN1b2.2 (NP_001266024.1) | MAVLKWLSICLTLFCQGTAASKPCRWTQFRLGKLNDVSIGLLSDMGGLFPLMCAEESVEQMFPEDLYKNTEGEDVYVVALEAMRYVEQLYNNSLTSVTWNKTKLNMFQNVIYRQVQNLELCVVGGVWESSGDGWSVTLKTYFNKLNTVLKEKEHSACAWEIVRKEIRENLVQFKKFIDSRVKL |
| >IFNIc2.4 (XP_014048249.1) | MALQTITWMSAFLCVAHVCSMPMPCQLQGQLVRITHNLLRDMGGNFPLDCLQENVFMAFPATAFASSGAPQLSSSGATAIYETLKNIDTLFGADDLPTQWDQQKLENFQNIVYRQIDESKCMMGSVDTSDYLIRTEGLNTYFGNIAAVLKEKNFSYCAWEVVRKELLYTLQFILEHNSDSLLWANRT |
| >IFNIa2.2 (XP_014048247.1) | MYTMQSWTCICLIICSMQSVCHCCDWIQHHYGHLSSEYLSLLDQMGGDITEQDAPVFFPTSLYRHIDDAEFEDQVRFRNETIYQITKLFDGNMKSVTWDKKKRDDFLNILERQFENLKSCVSPAKKPERRLKRYFKELNRKVLRKMNYSAQAWELIRKETRRHLQRLDIFKAKIH |
| >IFNIb2.3 (XP_014048072.1) | MAVLKWLSICLTLFCQGTAASKPCRWTQFRLGKLNDVSIGLLSDMGGLFPLMCAEENVEQMFPEDLYKNTEGEDVSVVALEAMRYVEQLYNNSLTSVTWNKTNLNMFQNVIYRQVQNLELCVVGGVWESSGDGWSVTLKTYFNKLNTVLKEKEHSACAWEIVRKEIRENLVQFKKFIDSRVKL |
| >IFNIe2.1 (XP_014047907.1) | MGSISLWMCLILTICTWNKTIGCTWMRTLPRSPSMFQVLSNNTITMLQKMGHVVSRKSQITFPNEQYRQVDNFTDNCRIVFISQTLNAIEKLYSSGKYDSTAWDQKGVDEFMIGLHRQTSELDQCVKTIKPGPSTSVKRVNKGMSLHFQILKNSLKLEEYSASGWEDIRNVVLSHMLRLVTIPID |
| >IFNIe2.2 (XP_014048073.1) | MGSISFWMCLVMTICTWNKTIGCTWMRTLPRSPSMFQVFSNNIITMLQKMGHEVSRDPQITFPDKQYRQVNNFKAEEQMAFISHTLNAIKKLYSSGKYESTAWDQKGVDKFMNDLYRQTSELDQCVKAMKTRLSKSVKRVNKKMSLHFKFLKNYLKREEYSASGWEDIRTVVLAHLQRLDTTLSSQ |
| >IFNIa2.3 (XP_014048070.1) | MYTMQSWTCIFLIICSMQSVCHCCDWIRHHYGHLSSEYLSLLDQMGGDITKQDAPVFFPRSLYRHIDDAEVEDQVRFLKETIYQITKLFDGNMKSVTWDKKNLDDFLNILERQLENLNSCVSPAMKPEKRLKRYFKKLNKNVLRKMNYSAQAWELIRKETKRHLQRLDILAAQMY |
| >IFNIa2.4 (XP_014048245.1) | MYTVQSWTCICLIICSMQSVCHCCDWIRHHYGHLSSEYLSLLDQMGGDITKQDAPVFFPTSLYRHIDDAEVEDKVRFLKETIYQITKLFDGNMKSVTWDKKKLDDFLNILERQLENLKSCVSPAMKPERRLKRYFKKLNKNVLRKMNYSAQAWELIRKETKRHLQRLDILAAQMY |
| >IFNIe2.3 (XP_014048244.1) | MGSISFWMCLVMTICTWNKTIGCTWMKTLPRSPSMFQVFSNNIITMLQKMGHEVSRDPQITFPDKQYRQVNNFKAEEQMAFISHTLNAIKKLYSSGKYESTAWDQKGVDKFMNDLYRQTSELDQCVKSMKTRLSKSVKRVNKKMSLHFKFLKNYLKREEYSASGWEDIRTVVLAHLQRLDTTLSSQ |
| >IFNIf2.1 (XP_014048243.1) | MGTLNLSFVVHLLCVIVLYPVVYAKCSDRNAQIYYLSQTRQTLNDLSMERRPRGCIPEAERLRVQRPTLSLQEGEKLWTLRLAFQLASELFQQNLTLVKWNSIKLRDLQDLLARQNMTYSECVRDMSVHLNLPIKNYFKRLDDFLLHERFSACSWEVVRAEMGSIISQVIKNAKKHV |
| >IFNIa1.1 (XP_014059915.1) | MYTMQSWTCLFLILCSMQSVCHCCEWIRHHFGHLSSEYLSQLDQMGGDITKQNAPVLFPTSLYRHIDDAEFEDKVRFLNETIYQIIKLFDGNMKSVTWDKKNLDDFLNILERQFENLNSCVSPAMKPESRLKRYFRKLNRKVLRKINYSAEAWELIRKETKRHLQRLDILAGQMY |
| >NP_001117042.1_interferon_alpha_2_precursor_Salmo_salar | MYTMQSWTCIFLIICSMQSVYHCCDWIRHHYGHLSSEYLSLLDQMGGDITKQDAPVFFPTSLYRHIDDGEVEDKVRFLKETIYQITKLFDGNMKSVTWDKKNLDDFLNILERQLENLNSCVSPAMKPERRLKRYFKKLNKNVLRKMNYSAQAWELIRKETKRHLQRLDILAAQMY |
| >IFNIc1.3 (XP_014060035.1) | MAIQMIIWMSAFLCLVQVFSMPMPCQLQGQLVRSTHNLLRDMGGHFPMECLQDNVFMEFPATAFATSGGPQLSSSGAKAIYETLKNIDTLFGTDELPTMWDQQKLEYFQNIVYRQIEESKCMMSSVDTSDYPIRAQGLKTYFGNIAAVLKEKKFSYCAWEVVRKELLYTLEFILKHNSDSLLWSNRT |
| >IFNIc1.2 (XP_014060034.1) | MAIQIIIWMSAFLCLVQVLSMPMPCQLKGQLVRTTQNLLRDMGGHFPVECLQDNVFMEFPATAFATSGGPQLSSSGAKALYETLKNIDTLFGTDELPTMWDQQKLEYFQNIVYRQIEESKCMMSSVDTSDYPIRAEGLKTYFGNIAAVLKEKNFSYCAWEVVRKELLYTLEFILKHNSDSLLWSNRT |
| >IFNIc1.1 (XP_014060031.1) | MAHQIIIWMSAFLCLVQVFSMPMPCQLQRHLVGTTYNLLRDMGGHFPLECLQDNNVFMVFPATAFATSGAPQLSSSGAKAIYETLKNIDTLFGTDELPTIWDQHKLEYFQNIIYRQMKESKCMMGSVNTRDYLIRAKMLNTYFRNIAAVLKEKNFSYCAWEVVRKELLYTLEFILKHNSDSLLWSNRT |
| >IFNId (NP_001266021.1) | MHRPTKSLLICLFLTMCDGFSMGCRWMDDHKFIQHSETLMNLLNIMGGEFTTDSVDVPFPEDLYKQAEYLPTDDTIWFILQTLDKIAELFDGEPDSVWNEKKVEIFLSVLNSQSDGLQSCVTAQKKNSKNLQMYFKRLHNQVLKRMAYSAHAWELVRKEVRTHLMRLVLLGSATENSI |
| >IFNIc2.1 (XP_014048254.1) | MALQTITWMSAFLCVAHVCSMPMPCQLQGQLVRITHNLLRDMGGNFPLDCLQENVFMAFPATAFASSGARQLSSSGAKAIYETLKNIDTLFGADDLPTQWDQRKLENFQNIVYRQIEESKCMMGSVDTSDYLIRTEGLNTYFGNIAAVLKEKNFSYCAWEVVRKELLYTLQFILEHNSDSLLWANRT |
| >IFNIb2.1 | MAVLKWLSICLTLFCQGTAASKPCRWTQFRLGNLNDVSIGLLSDMGGLFPLMCAEENVEQMFPEDLYKNTEGEDVYVVALEAMRYVEQLYNNSLTSVTWSKTELNMFQNVIYRQVQNLELCVVGGVWESSGNGWSVTLKTYFNKLNTVLKEKEHSACAWEIVRKEIRENLVQFKKFIDSRVKL |
| >IFNIc2.3 | MALQTITWMSAFLCVAHVCSMPMPCQLQGQLVRITHNLLRDMGGNFPLECLQENVFMAFPATAFASSGAPQLGSSGAKAIYETLKNIDILFEADDLPTQWDQQKLKNFQNIVYRQIEESKCMMGSVDTSDYLIRTEGLNTYFGNIAAVLKEKVDYRLNTYRHIVIYLCICLFMNVLIYLLENMLLFIKC |
| >IFNIf2.2 | MGTLNVSFVVHLLCVIVLYRVVYAKCSDQKEQMYYLSQTRQTLNYLSMRRPRGCIPEAERLRVQRPTLSLEEGEKLWTLRLAFQLASELFQQNLTLVKWNSIKLRDLQDLLARQNMTYSECVRDLRLRQNLVIGDMVKNYFKQLDDFLSREIKRYCFRSECTNSNNFVLYSDIGERNVCK |
| >IFNIf1.1 | MATLNVSFVVHLLCIIVLNPVVYAKCSDRNEQIYYLSRTRQTLNHLAMERMPSGCIQEAERIMVQRPTLSIEEGEKLWTRKLAFQLASELFKQNPTPVKWDTIKLRELQDLLARQNMTYSKCVRDMSVRLNLPIENMVKNYFKQLGDFLLHERFSSCSWEVVRDEMGRILRDFYKK |
| >NP_001117182.1_interferon_alpha_1_precursor_Salmo_salar | MYTVQSWTCICLIICSMQSVCHCCDWIRHHYGHLSSEYLSLLDQMGGDITKQDAPVFFPTSLYRHIDDAEVEDQVRFLKETIYQITKLFDGNMKSVTWDKKKLDDFLNILERQLENLKSCVSPAMKPEKRLKRYFKKLNKNVLRKMNYSAQAWELIRKETKRHLQRLDILAAQMY |
| >IFNIe1.1 | MGTSAFHFWNTLQNFCIKDRHRLEHETPPRSPSMFQVFSNNTITTLKKMGHKVSRESQITFPEKQYRQVDNLKADEQIAFILQTLNAIKKLYSSGKCESTSSNQKGVDKLMNDLYRQTSELDQCLRLSKSVKRVNKKMSLHFKFLKNYLKLAVYGASSWEDIRTVVLAHLQRLDMTLNSQ |
